# Assessing the Role of Shape and Label in the Misleading Packaging of Food Imitating Products: From Empirical Evidence to Policy Recommendation

**DOI:** 10.3389/fpsyg.2016.00450

**Published:** 2016-03-31

**Authors:** Frédéric Basso, Julien Bouillé, Kévin Le Goff, Philippe Robert-Demontrond, Olivier Oullier

**Affiliations:** ^1^Psychology, London School of Economics and Political ScienceLondon, UK; ^2^Faculty of Social Sciences—Department of Social and Economic Administration, University of Rennes 2 Upper BrittanyRennes, France; ^3^Cognitive Psychology Laboratory, Fédération de Recherche 3C, Centre National de la Recherche Scientifique, LPC UMR 7290, Aix Marseille UniversitéMarseille, France; ^4^Graduate School of Management, Center for Research in Economics and Management, UMR Centre National de la Recherche Scientifique 6211, University of Rennes 1Rennes, France

**Keywords:** category ambiguity, chemical consumer products, food package, Implicit Association Test (IAT), health policy, poison look-alikes

## Abstract

Food imitating products are chemical consumer items used frequently in the household for cleaning and personal hygiene (e.g., bleach, soap, and shampoo), which resemble food products. Their containers replicate elements of food package design such as possessing a shape close in style to drinking product containers or bearing labels that depict colorful fruits. In marketing, these incongruent forms are designed to increase the appeal of functional products, leading to chemical consumer product embellishment. However, due to the resulting visual ambiguity, food imitating products may expose consumers to the risk of being poisoned from ingestion. Thus, from a public health perspective, food imitating products are considered dangerous chemical products that should not be sold, and may merit being recalled for the safety of consumers. To help policymakers address the hazardous presence of food imitating products, the purpose of this article is to identify the specific design features that generate most ambiguity for the consumer, and therefore increase the likelihood of confusion with foodstuffs. Among the visual elements of food packaging, the two most important features (shape and label) are manipulated in a series of three lab studies combining six Implicit Association Tests (IATs) and two explicit measures on products' drinkability and safety. IATs were administered to assess consumers' implicit association of liquid products with tastiness in a within-subject design in which the participants (*N* = 122) were presented with two kinds of food imitating products with a drink shape or drink label compared with drinks (experiential products with congruent form) and classic chemical products (hygiene products) (functional products with congruent form). Results show that chemical consumer products with incongruent drink shapes (but not drink labels) as an element of food package design are both implicitly associated with tastiness and explicitly judged as safe and drinkable. These results require confirmation in other studies involving different shapes and labels. Notwithstanding, due to the misleading effect of this ambiguity, public health authorities are thus well advised to focus their market surveillance on chemical products emulating a food or drink shape.

## Introduction

This article highlights a widely used but surprisingly under-investigated marketing practice known as food imitating products. These are a multitude of chemical consumer products used daily for household cleaning and in personal hygiene routines (e.g., disinfectants, soaps, and shampoos). Their containers replicate elements of food package design, such as using labels that depict colorful fruits or possessing a shape that is deceptively fashioned like drinking products. Their incongruent form deviates from consumers' normative expectations (Noseworthy and Trudel, 2011) and thus food imitating products are deemed more attractive by looking tasty (Caraccio et al., 2006). Upon beholding these products, consumers are more likely to think automatically and positively about food than about an unpleasant chore (Basso et al., 2014). A concerning outcome is that elements of food package design obfuscate both the chemical nature of body care products (Herman, 2013) and the dangerous nature of household cleaners (Martens and Scott, 2005). Household products are usually flagged and sorted among the “toxic” and “unsafe” goods that are a source of modern health worries (Chen, 2013) and can be a cause of injury (Werner, 2003). They expose consumers and their children to potential hazards and accidental poisoning (Schneider, 1977). As food products imply the absence of impure additives and preservatives (Ger and Yenicioglu, 2004), food imitating products cultivate an image that suggests they are more natural than chemical, and less dangerous than regular chemical consumer products.

Paradoxically, their incongruent physical appearance may be implicated in an increased risk of poison through misuse. Food imitating features indeed bias how consumers process products. Corroborating evidence for this conclusion stems from reported exposure cases on specific chemical products on medical (e.g., Gould Soloway, 2008) or consumer advocacy websites (Harary, 2010), in the press (e.g., CNN, 2005; Bakalar, 2006; Bloom, 2007; Popken, 2008; Farrell, 2012) or in the scientific literature (Caraccio et al., 2006; Miller et al., 2006; Basso et al., 2014). From January to April 2006, 94 children and adults in the State of Texas mistakenly ingested *Fabuloso*^®;^, a cleaning product that both looks and smells like a drink (Miller et al., 2006; see also Caraccio et al., 2006). Since 2012, over 16,000 worldwide incidents involving children and adults who were injured by single-load liquid laundry packets resembling enticing candies were reported annually. This prompted an OECD global awareness-raising campaign on laundry detergent capsules involving 26 consumer product safety authorities from five continents (European Commission, 2015; see also Consumerunion.org, 2012; Fraser et al., 2012). Furthermore, many flipcharts, posters or videos on poison look-alikes released by regulatory authorities [e.g., (U.S. Consumer Product Safety Commission (CPSC), 2015; European Commission Health Consumers, 2013)], universities (e.g., The University of Kansas Hospital, 2012), poison control centers (e.g., The New Jersey Poison Control, 2014), or associations (e.g., Childsafetylink.ca., 2014; Safekids.nz, 2015) worldwide suggest that food imitating products are confusing.

In the present research, we suggest that food imitating products are ambiguous because they possess properties from two different categories (e.g., drinks and hygiene products), while consumers are accustomed to using only one category to classify products. This is coined the “single category belief problem” (Rajagopal and Burnkrant, 2009): consumers make single category (and thus biased) inferences in light of a product's physical form (Gregan-Paxton et al., 2005; Noseworthy et al., 2012). As such, food imitating products are chemical consumer products sharing physical features with drinks. They present as hybrid products and therefore lead to taste (i.e., biased) inferences.

To specify a product's physical form, the two most important visual features of food packaging, label (i.e., graphics and color), and shape (Silayoi and Speece, 2007), are selected and isolated. This creates two variations: Food imitating products with a drink shape and Food imitating products with a drink label. These two kinds of incongruent products are compared with prototypical drinks (experiential products with congruent form) and classic chemical consumer products (hygiene products; functional products with congruent form) in a lab experiment. Food imitating products are thus ambiguous if they can be categorized as drinks and as hygiene products.

Categorization ambiguity arising from perception of visual similarities is reasoned to be implicit (Noseworthy and Goode, 2011). Therefore, we assess the key elements causing food package design to bias consumers' implicit association of Food imitating products with tastiness via an Implicit Association Test (IAT; Greenwald et al., 1998). An IAT is a computer-based categorization task designed to approach implicit cognition by assessing the relative strengths of association among (target and attribute) concepts in memory. It achieves this without provoking introspection by using participants' reaction times to positive (pleasant) or negative (unpleasant) words or pictures. This methodological tool has successfully been used to study taste inferences (Raghunathan et al., 2006). At the explicit level, it is expected that food imitating products designed to appear more natural than chemical will be judged as safe and drinkable.

Across a series of three studies, this article tests whether food imitating products generate ambiguity through consumers' biased (taste) inferences and judgments on product safety and drinkability, and specify which incongruent product form is implicated in this effect; drink shape or drink label. In doing so, these results complement previous studies on children (Schwebel et al., 2015) and adults (Basso et al., 2014) and confirm the disquieting idea that the closer a chemical product resembles food, the more likely it is to be confused with food and subsequently swallowed (Scientific Committee on Consumer Safety, 2011; see also: Lueder and Rice, 2008; Basso et al., 2010). In addition, these results may indicate strategies for protection against this resulting heightened exposure to accidental poisonings, showing how psychology can facilitate policymakers in addressing the dark and dangerous side of chemical consumer product embellishment.

## Overview

Our main hypothesis is that Food imitating products are ambiguous products if they can be categorized as Drinks and as Hygiene products and judged as safe and drinkable (Studies #1 and #2). In each study, explicit measures of drinkability and safety were collected after participants completed two separate picture IAT experiments. In Study #1, participants thus completed a picture IAT where, compared to Drinks, Food imitating products with a drink label are paired with “untastiness” and are supposed to be categorized as Hygiene products; and another one where, compared to Hygiene products, Food imitating products with a drink shape are paired with “tastiness” and are supposed to be categorized as Drinks. Additionally, Food imitating products are anticipated to be judged as safe and drinkable, like Drinks and in contrast to Hygiene products. Study #2 focuses on the alternative comparisons. Involving two different studies to address our main hypothesis, this within-subject design ensures that participants are presented with the four existing different combinations of shape and label in each study, so that Drinks and Hygiene products can form reference points of what is drinkable and safe or not so, to discern if both kinds of Food imitating products are ambiguous at both implicit and explicit levels.

In a follow-up study (Study #3), we then examine which product form (drink shape or drink label) is the most ambiguous by testing whether shape or label of a drink has the greatest impact on consumers' biased (taste) inferences and judgments.

## Ethics statement

This study protocol is part of a larger research project on food imitating products that received the approval of local (Aix-Marseille Université Ethics Committee, France), regional (Comité de Protection des Personnes Sud Méditerranée 1, France) and national ethics, and regulatory agencies (French Agency for the Safety of Health Products/Agence Française de Sécurité Sanitaire des Produits de Santé, France). All volunteers gave their written informed consent prior to participating in these studies. Participants were debriefed at the end of their participation and each of them received a monetary compensation for his/her time.

## Study #1

In this first experiment, participants were expected to implicitly associate tastiness more strongly with Drinks than Food imitating products possessing a drink label (IAT 1A) and Food imitating products possessing a drink shape than Hygiene products (IAT 1B). In other words, picture IAT 1A tested if Food imitating products with a drink label can be categorized as Hygiene products when compared with Drinks, and picture IAT 1B tested if Food imitating products with a drink shape can be categorized as Drinks when compared with Hygiene products. Once the two separate picture IATs were completed, participants were asked to evaluate stimuli by answering the following questions: “*Would you say about each of the products depicted that it is drinkable(* = *1)/non-drinkable(* = *7) (or safe(* = *1)/dangerous(* = *7))?”* [see Batra and Ahtola (1991) on safe-dangerous scale] and were expected to explicitly evaluate Food imitating products as drinkable and safe.

### Method

#### Participants

Forty participants [*F* = 20, *M* = 20; *M*_age_ = 24.05, *SD* = 5.04; Body Mass Index (BMI): *M*_BMI_ = 22.25, *SD* = 2.6, normal or corrected-to-normal vision] recruited from Aix-Marseille University (France) were involved and completed the tasks in the same experimental setting (PC-type desktop, lab room) without the presence of other individuals including an experimenter.

#### Stimuli

There are two kinds of stimuli in a picture IAT experiment, target exemplars (pictures) and attribute exemplars (words). Twenty pictures of standardized Drinks, Hygiene products and Food imitating products served as exemplars of “Food” and “Hygiene” as target concepts, and ten food-related words served as exemplars of “Good” and “Bad” with respect to pleasant and unpleasant attribute concepts.

As an experiential product with congruent form, an exemplar of Drinks has two elements of food package design (drink label + drink shape) and, as a functional chemical product with congruent form, an exemplar of Hygiene product has, in theory, no element of food package design (hygiene product label + hygiene product shape). Rather, as a functional chemical product with an incongruent form, a Food imitating product either corresponds to an exemplar of a chemical product shaped like a drink (Food imitating product with a drink shape; hygiene product label + drink shape) or, conversely, corresponds to an exemplar of a chemical product labeled like a drink (Food imitating product with a drink label; hygiene product shape + drink label). Target concepts and their related exemplars across the three studies are reported in Table 1.

**Table 1:** **Target concepts and their related exemplars across the three studies**.

		**Target concepts**	
		**Food**	**Hygiene**
Study #1	IAT 1A	Exemplars of Drinks	Exemplars of Food imitating products with a drink label
	IAT 1B	Exemplars of Food imitating products with a drink shape	Exemplars of Hygiene products
Study #2	IAT 2A	Exemplars of Drinks	Exemplars of Food imitating products with a drink shape
	IAT 2B	Exemplars of Food imitating products with a drink label	Exemplars of Hygiene products
Study #3	IAT 3A	Exemplars of Food imitating products with a drink label	Exemplars of Food imitating products with a drink shape
	IAT 3B	Exemplars of Food imitating products with a drink shape	Exemplars of Food imitating products with a drink label

For this purpose, first of all, five shapes from drinks (*Coca-Cola*^®;^, *Orangina*^®;^, *Sprite*^®;^, *Joker*^®;^, *Evian*^®;^) were selected after 129 individuals (Female = 76, Male = 53; *M*_age_ = 20.4, *SD* = 1.36) answered unaided brand name recall questions. Given that consumers access a product category (e.g., Drinks) first through brands they use (Nedungadi and Hutchinson, 1985), an unaided brand name recall questionnaire was employed. Five shapes from exemplars of chemical products (*Milodor*^®;^, *Happy Shower*^®;^, *Visior*^®;^, *Javel Lacroix*^®;^, *Champion*^®;^ henna, and hazelnuts for brunettes) were selected from a qualitative field study on accidentally ingested chemical products among 31,283 medical records collected over a 14-month period at a Poison Control Center (see Basso et al., 2014 for details).

**Figure 1:**
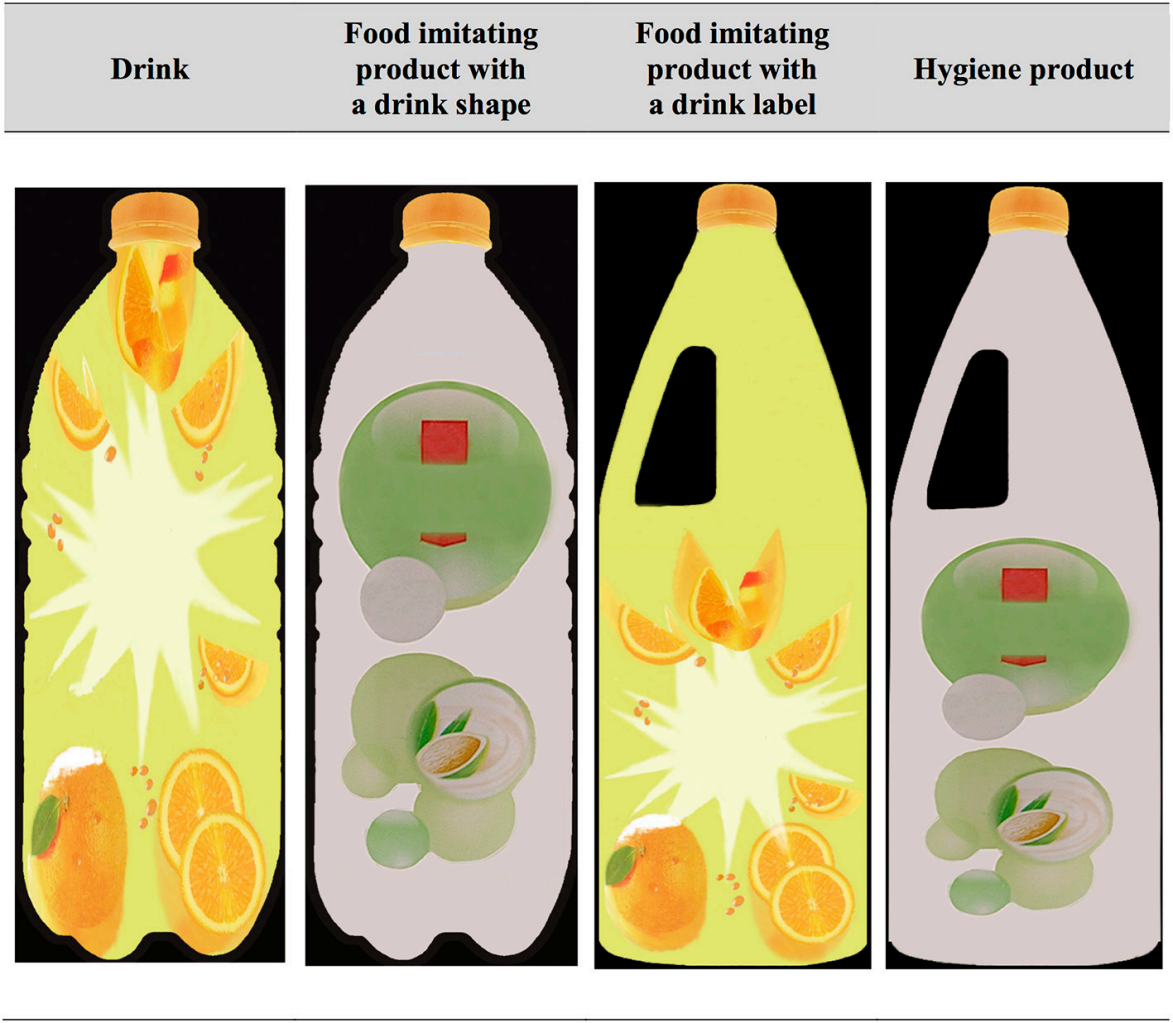
**Examples of target exemplars used in the experiment**. These are not actual items from the experiment due to copyright issues.

Then, drink and hygiene product labels were extracted from two very different food imitating products reported in a previous study (Basso et al., 2014). The drink label was extracted from the *Cottage Happy Shower*^®;^ *Tequila Sunrise*, a shower gel whose label is made up of images of oranges on a green background. The hygiene product label was extracted from the *Visior*^®;^ sweet almond cleaning product and is made up of one small image of an almond with green circles on a white background color. Unlike the *Visior*^®;^, *Happy Shower*^®;^ produced gustatory inferences in a neuroimaging (fMRI) experiment (see Basso et al., 2014 for details). The brand name on both labels were standardized (although keeping products' original typography) using the term “*Fabuloso*®,” the food imitating product accidentally ingested in the State of New York and in Texas (Caraccio et al., 2006; Miller et al., 2006). This brand name was selected since it remains unknown in France where the studies were conducted. Last, because of its impact on product perception, product size was controlled (e.g., Raghubir and Krishna, 1999). Figure 1 shows the type of stimuli used as target exemplars in picture IAT experiments.

To ensure that attribute exemplars appropriately defined the meaning of categories (Messner and Vosgerau, 2010), four specific controls on these words were made.

First, a list of French words synonyms of “Good” and “Bad” was generated on the website of the CRISCO lab at Caen University (France). Then, only synonyms that did not belong to the French National Education official list of the most frequent words used in French were kept. Words where the meaning was positive but where the form was negative (e.g., “inoffensive” as synonym of “Good”) and words written with an (acute or grave) accent for French speakers which are more difficult to understand in capital letters (e.g., “avéré” becomes “AVERE”) were removed from potential attribute exemplars. Last, eight independent judges (*F* = 4, *M* = 4; Age = 25−29) were asked to rate these words as synonyms of “Good” or “Bad” in the following statement: “*This orange juice is good (bad)*” (original *Happy Shower*^®;^ looking like an orange juice; see Basso et al., 2014, Medical case #F). The words rated as synonyms of “Good” or “Bad” with a similar frequency were selected by lot. Attribute exemplars are reported in Table 2.

**Table 2:** **Attribute exemplars used in the experiment (original French words and their respective frequencies are given in parentheses)**.

**Attribute exemplars**	
**Good (Positive attribute exemplars)**	**Bad (Negative attribute exemplars)**
Delicious (*Délicieux*; 0.875)	Bitter (*Amer*; 0.625)
Sweet (*Agréable*; 0.625)	Undrinkable (*Imbuvable*; 0.75)
Scrumptious (*Succulent*; 0.625)	Disgusting (*Infect*; 0.75)
Excellent (*Excellent*; 0.375)	Abominable (*Abominable*; 0.5)
Palatable (*Savoureux*; 0.375)	Mediocre (*Médiocre*; 0.5)

#### Apparatus and procedure

Picture IATs were programmed and administered with the INQUISIT 3 Milliseconds software package on a 15-inch computer screen. Participants entered responses using the left [E] and right [I] keys of the keyboard and were instructed to classify the items as quickly as possible. The computer recorded participants' response latencies in milliseconds.

As illustrated in Tables 3–**5**, picture IATs of the current study are examining a pair of target concepts—“Food” and “Hygiene”—and a pair of positive and negative attribute concepts—“Good” and “Bad.” More specifically, in light of Table 3 for instance, Picture IAT 1A examined the relative strengths of implicit associations of Drinks as target exemplars for “Food” and Food imitating products with a drink label as target exemplars for “Hygiene” with related words for “Good” or “Bad” as exemplars of positive and negative attribute concepts. Table 3 shows the response mappings in IATs across the Study #1.

**Table 3:** **Response mappings in IATs 1A and 1B**.

**Study #1**	**IAT 1A**		**IAT 1B**	
	**Left key [E]**	**Right key [I]**	**Left key [E]**	**Right key [I]**
Compatible blocks	Drinks + Good	Food imitating products with a drink label + Bad	Food imitating products with a drink shape + Good	Hygiene products + Bad
Incompatible blocks	Food imitating products with a drink label + Good	Drinks + Bad	Hygiene products + Good	Food imitating products with a drink shape + Bad

Picture IAT consists of seven blocks as the standard experimental protocol for IAT studies (Greenwald et al., 2003). Blocks 1, 2, and 5 are “practice blocks” so that participants are trained to the procedure. Blocks 3, 4, 6, and 7 are “measurement blocks” in which participants are randomly presented with one of the pictures or one of the positive (or negative) words.

In Block 1 of this categorization task, participants rapidly classified pictures of Drinks and Food imitating products with a drink label into the categories “Food” (by pressing the left computer key) and “Hygiene” (by pressing the right computer key). They then rapidly classified words into the categories “Good” and “Bad” (Block 2). In Block 3, the previous two tasks are combined in a compatible block of 20 trials and participants pressed the left computer key when any item in the category “Food” or “Good” appeared on the screen, and pressed the right computer key when any item in the category “Hygiene” or “Bad” appeared on the screen (i.e., the “Drinks + Good” or “Food imitating products with a drink label + Bad” pairing in IAT 1A). Block 4 repeats this procedure with an additional set of 40 trials. In the next stage (Block 5), the task in Block 1 is reversed and participants learned a new key mapping (Nosek et al., 2007). Similarly, Blocks 6 and 7 reverse the earlier combined pairings of Blocks 3 and 4 and constitute incompatible blocks: “Hygiene + Good” or “Food + Bad” (i.e., the “Food imitating products with a drink label + Good” or “Drinks + Bad” pairing in IAT 1A).

The difference in the latency to respond to particular pairings of target and attribute (here, “Drinks + Good” and “Food imitating products with a drink label + Bad”), compared to another set of pairings of target and attribute (“Food imitating products with a drink label + Good” and “Drinks + Bad”), provides an “index” of the relative strength of association with tastiness between the first vs. the second pairings (Lane et al., 2007).

In each study, to minimize the influence of order effects, the order of blocks is counterbalanced across participants and IATs so that half of participants did compatible trials first (the block sequence 1–2–3–4–5–6–7) and the other half did incompatible trials first (the block sequence 1–2–6–7–5–3–4). In each block, the trials are presented in a novel random order for each participant. The order in which participants did the two IAT experiments, and the number of participants doing compatible or incompatible blocks first was counterbalanced. Positive and negative words as attribute stimuli were written in blue (RGB 0 235 243) instead of green (RGB 0 255 0) as drink label was green and might have interfered with participants classification latencies. When participants pressed the wrong key, a red cross appeared centrally in red, and remained on the display until the correct key was pressed.

#### Measures

The response latencies in measurement blocks served as the basis for calculating participants' automatic associations. An “inclusive” standard deviation for all trials in Blocks 3 and 6 and for all trials in Blocks 4 and 7 is computed. In addition, the mean latency for responses of each Block 3, 4, 6, and 7 is computed. The two mean differences (M_Block6_–M_Block3_) and (M_Block7_–M_Block4_) are calculated and each difference score is divided by its associated “inclusive” standard deviation. The *D* score is the equal-weight average of two resulting ratios. When responses are significantly faster in the compatible blocks than in the incompatible blocks, a positive *D* score is interpreted as revealing a stronger automatic association with positive attribute exemplars (words for “tastiness”) of target exemplars from compatible blocks (e.g., Drinks in IAT 1A) than of target exemplars from incompatible blocks (e.g., Food imitating products with a drink label) (e.g., Brunel et al., 2004). IAT *D* scores were calculated by using the improved algorithm that requires a minimum sample size of 39 participants (Greenwald et al., 2003). Participants (none) were excluded from the analyses if 10% of his/her latencies were shorter than 300 ms. Participant response latencies longer than 10,000 ms (none) and shorter than 300 ms (six out of 9600 critical trials) were removed from the analyses in Study #1.

Stimuli drinkability (safety) is calculated in each study as the average for a category of products of the means of the evaluation of each product on drinkable/non-drinkable (safe/dangerous) scale by each participant in the study. For instance, in Study #1, Drinks drinkability (safety) is the average of the means of the evaluation of the five exemplars of Drinks on the drinkable/non-drinkable (safe/dangerous) scale by each of the participants of this study. The scores on the drinkable/non-drinkable and the safe/dangerous scales ranged from 1 (= drinkable; or = safe) to 7 (= non-drinkable; or = dangerous) with four as the midpoint of the scale. A product was considered to be drinkable or safe when the mean scale score was significantly below the scale midpoint.

### Analysis

We conducted planned contrasts using two-tailed paired Student's *t*-tests to compare the means of response latencies related to compatible and incompatible blocks, and two-tailed one sample Student's *t*-test against the respective scale midpoint. Additional two-tailed paired Student's *t*-tests compared Food imitating products and Drinks (Hygiene products) scores on the drinkable/non-drinkable (safe/dangerous) scale. All statistical analyses were performed with SPSS version 22 (SPSS Inc., Chicago, IL).

### Results

**Figure 2:**
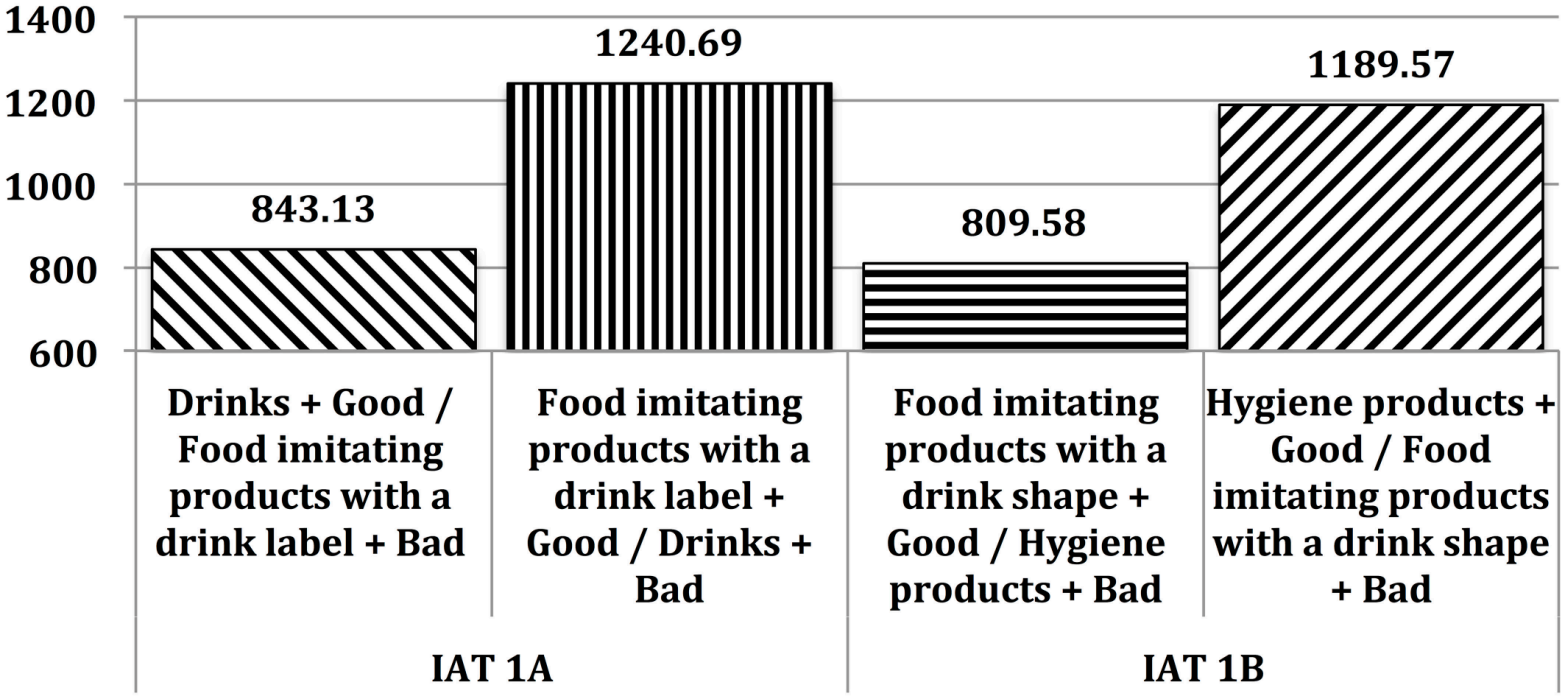
**Mean response latencies (in milliseconds) in Study #1**.

At the implicit level, tastiness was more strongly associated with Drinks than with Food imitating products possessing a drink label [IAT 1A *D* effect = 0.81; *M*_IAT_1A_Compatible_ = 843.13*ms*; *M*_IAT_1A_Incompatible_ = 1240.69*ms*; *t*_(39)_ = 11.20; *p* < 0.000]. This tastiness association was also stronger with Food imitating products possessing a drink shape than with Hygiene products [IAT 1B *D* effect = 0.79; *M*_IAT_1B_Compatible_ = 809.58*ms*; *M*_IAT_1B_Incompatible_ = 1189.57*ms*; *t*_(39)_ = 9.04; *p* < 0.000]. Response latencies are reported in Figure 2.

At the explicit level, Drinks [*M*_Drinkability_ = 1.36, *SD* = 0.67, *t*_(39)_ = −24.84; *p* < 0.000; *M*_Safety_ = 1.83, *SD* = 0.85, *t*_(39)_ = −16.09; *p* < 0.000] and Food imitating products with a drink shape [*M*_Drinkability_ = 2.38, *SD* = 1.75, *t*_(39)_ = −5.85; *p* < 0.000; *M*_Safety_ = 2.47, *SD* = 1.25, *t*_(39)_ = −7.72; *p* < 0.000] were evaluated as drinkable and safe. Food imitating products with a drink label [*M*_Drinkability_ = 6.345, *SD* = 1.08, *t*_(39)_ = 13.76; *p* < 0.000; *M*_Safety_ = 4.33, *SD* = 1.29, *t*_(39)_ = 1.64; *p* = 0.108] and Hygiene products [*M*_Drinkability_ = 6.78, *SD* = 0.59, *t*_(39)_ = 29.53; *p* < 0.000; *M*_Safety_ = 4.48, *SD* = 1.53, *t*_(39)_ = 2.00; *p* = 0.052] were not evaluated as drinkable and safe.

Food imitating products with a drink shape [*t*_(39)_ = 14.857; *p* < 0.000] and Food imitating products with a drink label [*t*_(39)_ = 3.475; *p* < 0.001] were both significantly evaluated as more drinkable than Hygiene products. But neither Food imitating products with a drink shape [*t*_(39)_ = −3.95; *p* < 0.000] nor Food imitating products with a drink label [*t*_(39)_ = −21.17; *p* < 0.000] were evaluated as drinkable as Drinks.

Food imitating products with a drink shape [*t*_(39)_ = 7.73; *p* < 0.000], but not Food imitating products with a drink label [*t*_(39)_ = 1.03; *p* = 0.311], were significantly evaluated as less dangerous than Hygiene products.

## Study #2

This second study focused on the alternative comparisons between Drinks, Hygiene products and both kinds of Food imitating products. Picture IAT 2A tested if Food imitating products with a drink shape can be categorized as Hygiene products when compared with Drinks, and picture IAT 2B tested if Food imitating products with a drink label can be categorized as Drinks when compared with Hygiene products. Participants were thus expected to implicitly associate tastiness more strongly with Drinks than Food imitating products possessing a drink shape (IAT 2A) and with Food imitating products possessing a drink label than Hygiene products (IAT 2B). They were also expected to explicitly evaluate Food imitating products as drinkable and safe.

### Method

#### Participants

Forty-one participants (*F* = 21, *M* = 20; *M*_age_ = 22.29, *SD* = 1.68; *M*_BMI_ = 21.21, *SD* = 2.54, normal or corrected-to-normal vision) recruited from the University of Rennes 1 (France) and different from those who participated in Study #1, were involved and completed the tasks in a manner similar to Study #1.

#### Stimuli

Stimuli are similar to those used in Study #1.

#### Apparatus and procedure

Apparatus and procedure are similar to those used in Study #1. Table 4 shows the response mappings in IATs across the Study #2.

**Table 4:** **Response mappings in IATs 2A and 2B**.

**Study #2**	**IAT 2A**		**IAT 2B**	
	**Left key [E]**	**Right key [I]**	**Left key [E]**	**Right key [I]**
Compatible blocks	Drinks + Good	Food imitating products with a drink shape + Bad	Food imitating products with a drink label + Good	Hygiene products + Bad
Incompatible blocks	Food imitating products with a drink shape + Good	Drinks + Bad	Hygiene products + Good	Food imitating products with a drink label + Bad

#### Measures

Six out of 9840 critical trials shorter than 300 ms and seven out of 9840 critical trials longer than 10,000 ms were removed from the data analyses in Study #2.

#### Analysis

Data analysis was similar to Study #1.

### Results

**Figure 3:**
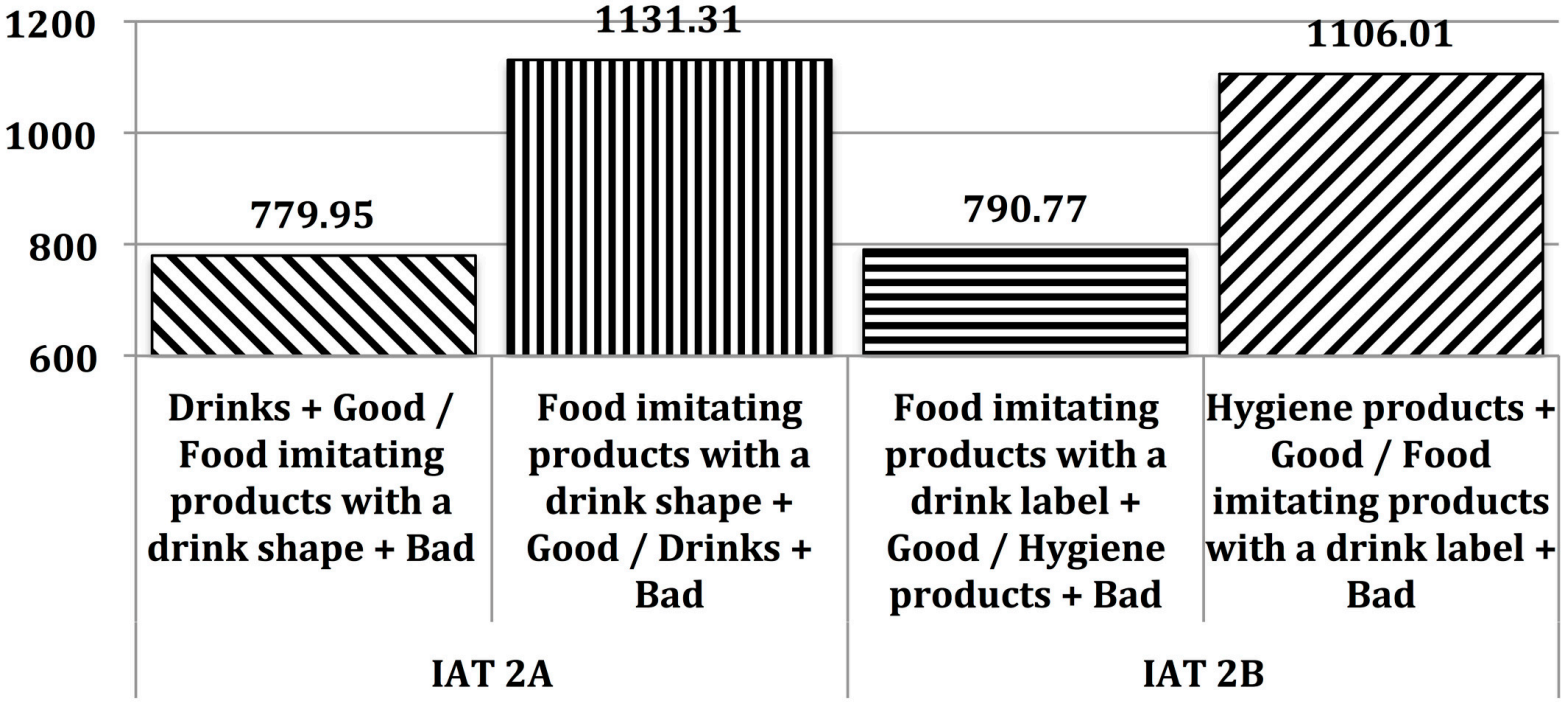
**Mean response latencies (in milliseconds) in Study #2**.

At the implicit level, tastiness was more strongly associated with Drinks than with Food imitating products possessing a drink shape [IAT 2A *D* effect = 0.70; *M*_IAT_2A_Compatible_ = 779.95*ms*; *M*_IAT_2A_Incompatible_ = 1113.31 ms; *t*_(40)_ = 7.28; *p* < 0.000]. This tastiness association was also stronger with Food imitating products possessing a drink label than with Hygiene products [IAT 2B *D* effect = 0.65; *M*_IAT_2B_Compatible_ = 790.77*ms*; *M*_IAT_2B_Incompatible_ = 1106.01*ms*; *t*_(40)_ = 8.48; *p* < 0.000]. Response latencies are reported in Figure 3.

At the explicit level, Drinks [*M*_Drinkability_ = 1.42, *SD* = 0.48, *t*_(40)_ = −34.63; *p* < 0.000; *M*_Safety_ = 1.58, *SD* = 0.60, *t*_(40)_ = −25.98; *p* < 0.000] were evaluated as drinkable and safe. Food imitating products with a drink shape [*M*_Drinkability_ = 5.22, *SD* = 1.61, *t*_(40)_ = 4.89; *p* < 0.000; *M*_Safety_ = 4.50, *SD* = 1.58, *t*_(40)_ = 2.04; *p* < 0.048], Food imitating products with a drink label [*M*_Drinkability_ = 4.86, *SD* = 1.53, *t*_(40)_ = 3.60; *p* < 0.001; *M*_Safety_ = 4.34, *SD* = 1.25, *t*_(40)_ = 1.73; *p* = 0.092] and Hygiene products [*M*_Drinkability_ = 6.85, *SD* = 0.25, *t*_(40)_ = 73.52; *p* < 0.000; *M*_Safety_ = 4.96, *SD* = 1.55, *t*_(40)_ = 3.96; *p* < 0.000] were not evaluated as drinkable and safe.

Food imitating products with a drink shape [*t*_(40)_ = 6.57; *p* < 0.000] and Food imitating products with a drink label [*t*_(40)_ = 8.23; *p* < 0.000] were both significantly evaluated as more drinkable than Hygiene products. But neither Food imitating products with a drink shape [*t*_(40)_ = −14.41; *p* < 0.000] nor Food imitating products with a drink label [*t*_(40)_ = −13.95; *p* < 0.000] were evaluated as drinkable as Drinks.

Food imitating products with a drink label [*t*_(40)_ = 2.99; *p* < 0.005], but not Food imitating products with a drink shape [*t*_(40)_ = 1.66; *p* = 0.104], were significantly evaluated as less dangerous than Hygiene products.

## Study #3

In light of Studies #1 and #2, both kinds of Food imitating products are evaluated as more drinkable than Hygiene products and can be categorized as Drinks (or as Hygiene products) when compared with Hygiene products (or Drinks) in a picture IAT experiment. Interestingly, Food imitating products with a drink shape were significantly evaluated as less dangerous than Hygiene products when implicitly associated with tastiness (Study #1), and similarly for Food imitating products with a drink label (Study #2). However, while Food imitating products with a drink shape were evaluated as safe and drinkable (though not as drinkable as Drinks) at the explicit level when associated with tastiness at the implicit level (Study #1 IAT 1B), this does not occur in the case of Food imitating products with a drink label (Studies #1 IAT 1A and #2 IAT 2B). Therefore, Food imitating products with a drink shape seem to be more ambiguous than Food imitating products with a drink label.

In Study #3, participants were presented with the two kinds of Food imitating products as incongruent hygiene products to examine which product form is the most ambiguous.

Picture IAT 3A examined the relative strengths of implicit associations of Food imitating products with a drink label as paired with positive attribute concepts (“Good”) and Food imitating products with a drink shape as paired with negative attribute concepts (“Bad”). This means that Food imitating products with a drink label were supposed to be compatible with positive words for tastiness (in Block 1, participants learned that Food imitating products with a drink label were exemplars of Food) and Food imitating products with a drink shape were supposed to be incompatible with positive words for tastiness (in Block 1, participants learned that Food imitating products with a drink shape were exemplars of Hygiene).

Conversely, picture IAT 3B examined the relative strengths of implicit associations of Food imitating products with a drink shape as paired with positive attribute concepts (“Good”) and Food imitating products with a drink label as paired with negative attribute concepts (“Bad”). This means that Food imitating products with a drink shape were supposed to be compatible with positive words for tastiness (in Block 1, participants learned that Food imitating products with a drink shape were exemplars of Food) and Food imitating products with a drink label were supposed to be incompatible with positive words for tastiness (in Block 1, participants learned that Food imitating products with a drink label were exemplars of Hygiene).

### Method

#### Participants

Forty-one participants (*F* = 21, *M* = 20; *M*_age_ = 21.46, *SD* = 1.45; *M*_BMI_ = 21.75, *SD* = 2.44, normal or corrected-to-normal vision), recruited from the University of Rennes 1 (France) and the University of Rennes 2 Upper Brittany (France) and different from those who participated in Studies #1 and #2, were involved and completed the tasks in a manner similar to Study #1.

#### Stimuli

Stimuli are similar to those used in Study #1.

#### Apparatus and procedure

Apparatus and procedure are similar to those used in Study #1. Table 5 shows the response mappings in IATs across the Study #3.

**Table 5:** **Response mappings in IATs 3A and 3B**.

**Study #3**	**IAT 3A**		**IAT 3B**	
	**Left key [E]**	**Right key [I]**	**Left key [E]**	**Right key [I]**
Compatible blocks	Food imitating products with a drink label + Good	Food imitating products with a drink shape + Bad	Food imitating products with a drink shape + Good	Food imitating products with a drink label + Bad
Incompatible blocks	Food imitating products with a drink shape + Good	Food imitating products with a drink label + Bad	Food imitating products with a drink label + Good	Food imitating products with a drink shape + Bad

#### Measures

One out of 9840 critical trials shorter than 300 ms and one out of 9840 critical trials longer than 10,000 ms were removed from the computation of the IAT *D* effect in Study #3.

#### Analysis

Data analysis was similar to Study #1.

### Results

**Figure 4:**
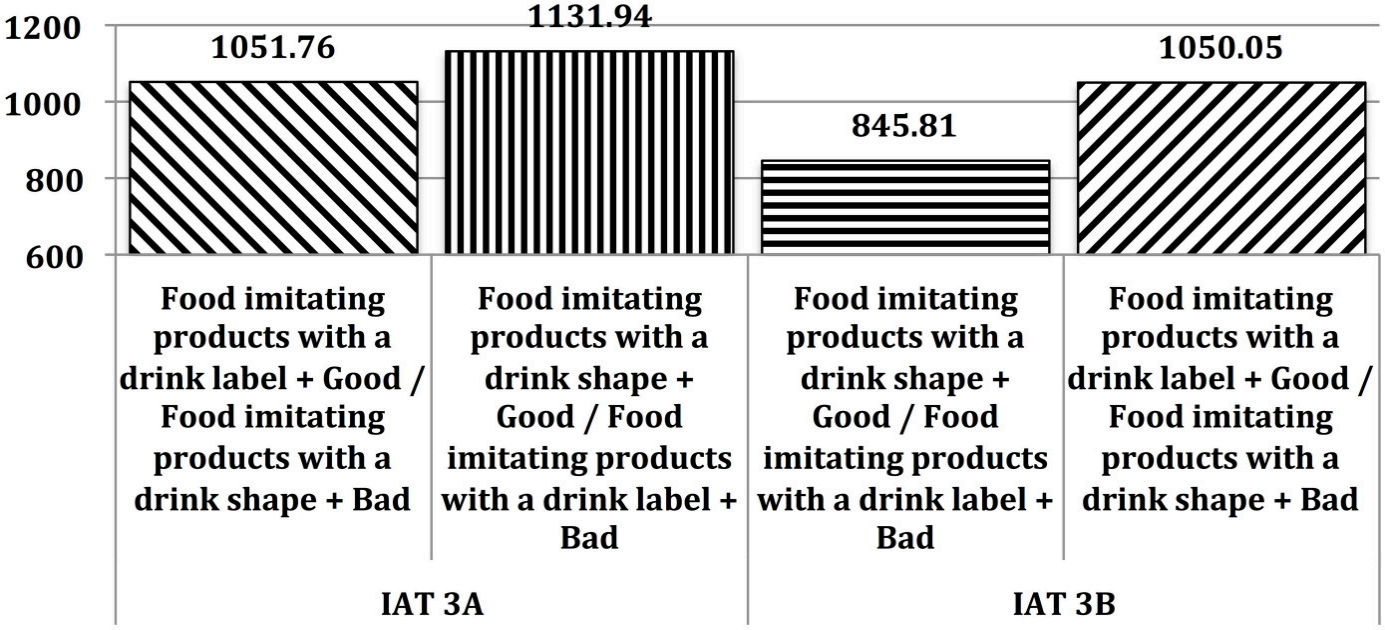
**Mean response latencies (in milliseconds) in Study #3**.

At the implicit level, tastiness was not more strongly associated with Food imitating products possessing a drink label than with Food imitating products with a drink shape [IAT 3A *D* effect = 0.13; *M*_IAT_3A_Compatible_ = 1051.76*ms*; *M*_IAT_3A_Incompatible_ = 1131.94*ms*; *t*_(40)_ = 1.25; *p* = 0.22] but was more strongly associated with Food imitating products possessing a drink shape than Food imitating products possessing a drink label [IAT 3B *D* effect = 0.44; *M*_IAT_3B_Compatible_ = 845.81*ms*; *M*_IAT_3B_Incompatible_ = 1050.05*ms*; *t*_(40)_ = 4.2; *p* < 0.000]. Response latencies are reported in Figure 4.

At the explicit level, Drinks [*M*_Drinkability_ = 1.38, *SD* = 0.57, *t*_(40)_ = −29.33; *p* < 0.000; *M*_Safety_ = 1.75, *SD* = 0.76, *t*_(40)_ = −18.85; *p* < 0.000] and Food imitating products with a drink shape [*M*_Drinkability_ = 3.08, *SD* = 2.01, *t*_(40)_ = −2.93; *p* < 0.006; *M*_Safety_ = 2.8, *SD* = 1.36, *t*_(40)_ = −5.65; *p* < 0.000] were evaluated as drinkable and safe. Food imitating products with a drink label [*M*_Drinkability_ = 6.23, *SD* = 0.97, *t*_(40)_ = 14.804; *p* < 0.000; *M*_Safety_ = 4.35, *SD* = 1.40, *t*_(40)_ = 1.61; *p* < 0.116] and Hygiene products [*M*_Drinkability_ = 6.70, *SD* = 0.68, *t*_(40)_ = 25.24; *p* < 0.000; *M*_Safety_ = 4.55, *SD* = 1.50, *t*_(40)_ = 2.33; *p* < 0.025] were not evaluated as drinkable and safe.

Food imitating products with a drink shape [*t*_(40)_ = 10.61; *p* < 0.000] and Food imitating products with a drink label [*t*_(40)_ = 3.62; *p* < 0.001] were both significantly evaluated as more drinkable than Hygiene products. But neither Food imitating products with a drink shape [*t*_(40)_ = −5.47; *p* < 0.000] nor Food imitating products with a drink label [*t*_(40)_ = −23.09; *p* < 0.000] were evaluated as drinkable as Drinks.

Food imitating products with a drink shape [*t*_(40)_ = 5.32; *p* < 0.000], but not Food imitating products with a drink label [*t*_(40)_ = 1.93; *p* = 0.06], were significantly evaluated as less dangerous than Hygiene products.

## Discussion

Our results strongly align with the posited idea that product form incongruity can call product functionality into question (Noseworthy and Trudel, 2011) and illustrate the danger of *inaccurate* product *embellishment* (Alba and Hutchinson, 1987; Hoegg and Alba, 2008). Through the “single category belief problem” (Rajagopal and Burnkrant, 2009), this article clearly illustrates the extent to which elements of food package design are ambiguous and impact the judgment of consumers on (incongruent) chemical products. This is consistent with studies in human factors research, which have shown how adults may make assumptions about the hazard levels of products based on the physical characteristics of the container (Wogalter et al., 1997). A caveat regarding biased or erroneous judgments is important; if a manufacturer fills a container with a more dangerous chemical than consumers expect it to contain, consumers may be misled by product appearance and underestimate the health risk at hand (Serig, 2000).

Across Studies #1 and #2, as expressed by IAT *D* effects in IATs 1B and 2B, regardless as to whether these Food imitating products were labeled or shaped like a drink, participants consistently more strongly associated them with tastiness than Hygiene products. Elements of food package design lead participants to think automatically and positively about food while looking at exemplars of chemical products, which might explain why food imitating products are an increasingly widespread marketing practice for chemical products that biases consumer judgment.

Although both kinds of Food imitating products can be categorized as Hygiene products (IATs 1A and 2A) and as Drinks (IATs 1B and 2B), neither Food imitating products with a drink shape nor Food imitating products with a drink label were evaluated as drinkable as Drinks in Studies #1 and #2. It is likely however that, in these studies, explicit attitudes have been influenced by prior implicit measures. Indeed, when Food imitating products were implicitly associated with tastiness in IATs 1B and 2B, they were then evaluated as more drinkable and less dangerous than Hygiene products. Moreover, Food imitating products with a drink shape were evaluated as safe and drinkable after participants completed IAT 1B.

Study #3 ensured that the explicit evaluations of both kinds of Food imitating products were equally influenced by IATs that come prior to them. In IAT 3A, participants learned to categorize Food imitating products with a drink label as exemplars of “Food” and Food imitating products with a drink shape as exemplars of “Hygiene.” Conversely, in IAT 3B, they learned to categorize Food imitating products with a drink shape as exemplars of “Food” and Food imitating products with a drink label as exemplars of “Hygiene.”

Interestingly, the observed IAT effects in Study #3 reveal that participants did not associate Food imitating products possessing a drink label with tastiness and Food imitating products possessing a drink shape with “untastiness” (IAT 3A) but did associate Food imitating products possessing a drink shape with tastiness and Food imitating products possessing a drink label with “untastiness” (IAT 3B). This means that participants more strongly associated tastiness with Food imitating products possessing a drink shape than Food imitating products possessing a drink label. At the explicit level and similarly to Study #1, Food imitating products with a drink shape were evaluated as drinkable and safe contrary to Food imitating products with a drink label and Hygiene products. To concisely summarize, Food imitating products with a drink shape emerge as most ambiguous; more so than Food imitating products with a drink label and Hygiene products.

The majority of the hypotheses are supported by the empirical findings but these conclusions come with limitations that indicate avenues for future research. First, we cannot exclude that the drink shapes tested were likely to be more associated with tastiness than the drink label tested. Indeed, the drink shapes were based on the product shapes of five drinks generated through an unaided brand name recall task whereas the drink label used was not from drinks but extracted from a food imitating product, the *Cottage Happy Shower*^®;^ *Tequila Sunrise*. Further investigation manipulating real drink labels, such as those of the five drink shapes used in the current studies, would illuminate whether drink shapes or labels lead to greater product ambiguity in household products. Second, while participants did not report that the almond label of *Visior*^®;^ belonged to a drink when they had been debriefed at the end of the IATs, it would be interesting to show in future studies that even subtle references to food, such as through food pictures or related colors in the label on food-like containers, is confusing to consumers. Third, these studies were conducted on adult student participants and it is implicitly assumed that food imitating products causing confusion for adults will *ipso facto* be confusing for the most exposed and poisoned demographic; children (Basso et al., 2014). Recent experimental studies show that children are likely to consider shape, size, labeling, and color when deciding whether a product is edible/drinkable in order to avoid incurring foreseeable injury (Schwebel et al., 2015). Future studies seeking to administer the IAT to children (Baron and Banaji, 2006) should therefore be adapted to test the generalizability of our findings. Last, this study focuses on food imitating products in the context of showing how drink shape or label biases chemical consumer product judgment. However, there are also other famous cases of chemical products that are sold such as beauty products (e.g., *Meyer's Clean Day*^®;^ line; Chaker, 2011) or designer collaboration product lines (e.g., *Mir*^®;^ *Graphic Egery* dishwashing liquid collection; see also, Kirwan-Taylor, 2003). More importantly, the marketing of play as a consumption experience (Holbrook et al., 1984) also inspires the introduction of new chemical consumer products on the market (e.g., *Bref Freshsurfer*^®;^ toilet block; see also, Scientific Committee on Consumer Safety, 2011). Future research could explore these multiple and emerging frontiers of food imitating product design to examine if chemical consumer products which feature child-appealing properties bias product judgment as well.

## Policy thinking and applications

This article reveals the extent to which consumers' perception and judgments are being fooled by food imitating products, and how chemical consumer product embellishment represents and poses a very serious danger. One must acknowledge that poisoning might be the result of “a poor use of ‘safe’ products” (Staelin, 1978, p. 30) or of individual (host) and/or contextual influences (McFarland, 1955). However, when a product appears safer and better than it is in reality, it could be used in an unconventional and possibly dangerous manner (see Wansink, 2003). This is why, in accordance with the European Council Directive 87/357/EEC, food imitating products are considered dangerous chemical products that should not be sold and could qualify for being recalled in the name of consumer safety.

The Poison Packaging Prevention Act (PPPA) of 1970 (15 U.S.C. § 1472–1473) sought to reduce child-poisoning rates in the United States by prohibiting packages deemed “unnecessarily attractive to children” and mandated a child-resistant packaging on hazardous household products (“hazardous substances”). Despite this regulatory framework, food imitating products are pervasive in the U.S. marketplace. This has led to unintentional ingestion exposures not only among children but an adult cohort too. According to data from the U.S. Consumer Product Safety Commission (CPSC) (2005), from 1991 to 2002, the most noteworthy injury pattern for older adult consumers entailed failing to recognize the container of a household chemical product or mistaking it for another product.

There are more stringent rules in the European Union where the Council Directive 87/357/EEC (article #1) prohibits the marketing, import and either manufacture or export of products that although not foodstuffs, possess a form, odor, color, appearance, packaging, labeling, volume, or size that is confusing. From a policymaking perspective, the scope of the European Council Directive 87/357/EEC is not limited to chemical products and is wider than the Poison Packaging Prevention Act (PPPA) of 1970. More interestingly, for European Member States being required to withdraw any food imitating product from their markets (article #3), the General Product Safety Directive (2001/95/EC) launched the RAPEX in 2001; a rapid alert system for non-food consumer products. When a European Member State detects a product that is unsafe/dangerous, the European Commission is informed and, after validation, information is circulated in all Member States participating in the system (Directorate General for Health Consumers, 2010, Figure 40). Over the last 10 years (2005–2015), the RAPEX facilitated the withdrawal of 258 dangerous products from the European market, preventing further risks to consumers.

However, while the Poison Prevention Packaging Act (PPPA) remains evasive regarding the packages “unnecessarily attractive to children” and can be circumvented (Consumerreports.org, 2006; Anthony, 2011), the current European regulatory framework gives both too much and too little guidance. The criteria listed in the aforementioned legislations virtually encompass all the food-packaging elements, but do not delineate any specific method for assessing food imitating products: any chemical consumer product out of the thousands that are sold on a given market should be screened and withdrawn when there is one food-packaging element. As proposed by the Directorate General for Health Consumers (2011) of the European Commission that manages the RAPEX, a standardized approach to the assessment of chemical consumer products' safety across the European Union is needed. However, ranking key elements of a package design that are likely to increase the probability for confusion with foodstuffs was not deemed possible in light of the absence of available poisoning data to inform an answering of this question (Scientific Committee on Consumer Safety, 2011). The scientific literature substantiates the assertion that public policy should encourage manufacturers to develop chemical consumer products labeled with symbols children can recognize (e.g., unappealing insects), in the upstream market. Opaque and squared packaging is preferable to the transparent and rounded variety, and should be made up of metal rather than plastic materials (Schwebel et al., 2015). In the downstream market, our results can aid public health policy efforts in several markets to address post-purchase food imitating products by focusing their market surveillance on chemical consumer products with a food or drink shape.

Although these results require additional study confirmation, the current research can help to address the need for a unified approach to the assessment of chemical consumer products' safety across the European Union. It provides a timely examination of the product features that contribute to the confusing nature of food imitating products in the context of the revision of the existing legislations in Europe (see Silbergeld et al., 2015 on the challenges of regulating industrial chemical products in the United States in light of the European Union legislation).

Last, although the U.S. Consumer Product Safety Commission (CPSC) is not supposed to prescribe specific packaging designs [Poison Packaging Prevention Act (PPPA) of 1970, 15 U.S.C. § 1472], petitions can be addressed to the Commission as recently illustrated by the request for non-see-through packaging for torch fuel and lamp oil resembling juice that resulted in accidental ingestion by children and adults [U.S. Consumer Product Safety Commission (CPSC), Docket No. CPSC-2011-0048]. Our results could thus be used in consumer advocacy against food imitating products (Bouillé et al., 2014).

## Conclusion

In previous publications on food imitating products (Basso et al., 2010, 2014), we employed several psychological methodologies ranging from the analysis of naturally occurring talk to behavioral and functional neuroimaging experiments to support evidence-informed policymaking (Oullier, 2013). We think that an additional benefit of the studies reported in this article is the provision of significant public policy insights that identify how consumers would react to dangerous marketing strategies that can potentially harm them. It also shows how experimental psychology methods (such as the IAT) can help public authorities enforce manufacturers of chemical products to run rigorous tests that will determine the propensity of people to misjudge products, and therefore make an evidence-informed decision regarding their market release (or withdrawal). In addition, given that implicit attitudes could exert more influence when consumers are distracted or making quick decisions (e.g., when consumers inadvertently drink a chemical product; Gibson, 2008), policymakers should concentrate their market surveillance on chemical consumer products with a drink shape; a prime design feature shown to bias consumers' (chemical) product judgment and to expose them to the risk of being poisoned.

## Author contributions

Conceived and designed the experiments: FB, JB. Performed the experiments: FB, JB, KLG. Analysed the data: FB, JB. Contributed reagents/materials/analysis tools: FB, JB, KLG. Wrote the paper: FB and JB helped by OO, PRD. Revised the manuscript: FB.

## Funding

This work was funded by Aix Marseille Université (Marseille, France), University Rennes 1 (Rennes, France) and the London School of Economics and Political Science (London, UK).

### Conflict of interest statement

The authors declare that the research was conducted in the absence of any commercial or financial relationships that could be construed as a potential conflict of interest.
